# Population Density and Spatial–Temporal Activity Pattern of the Tibetan Wolf in Dulan, Qinghai, China

**DOI:** 10.3390/biology14091273

**Published:** 2025-09-16

**Authors:** Liulin Guan, Liping Tan, Junchen Liu, Xinyang Chen, Shanshan Zhao, Guosheng Wu, Yonghong Shi, Xiao Song, Aichun Xu

**Affiliations:** 1 College of Life Sciences, China Jiliang University, Hangzhou 310018, China; 2 Xining Wildlife Park, Xining 810008, China; 3 Dulan County Forestry and Grassland Bureau, Haixi 816199, China

**Keywords:** wildlife conservation, random encounter model, Qinghai–Tibetan Plateau, camera traps

## Abstract

As apex predators, wolves (*Canis lupus*) play a vital role in balancing and stabilizing ecosystems. However, the scarcity of empirical data on the population size and spatial ecology of wolves in Qinghai Province, China, poses significant challenges to the design of targeted conservation measures, with cascading effects on both regional ecosystem integrity and the long-term viability of this ecologically critical species. To address this knowledge gap, we deployed a systematic grid of 150 camera traps across Dulan County, Qinghai Province, from July 2021 to July 2022. The survey effort totaled 41,610 camera-days, yielding 392 independent wolf detections, defined as events separated by ≥24 h at the same location. The random encounter model estimated wolf density across three habitat types in Dulan County as follows: alpine meadows (4.87 ± 0.87 individuals/100 km^2^), bare rock areas (21.39 ± 3.79 individuals/100 km^2^), and alpine shrub (7.81 ± 1.31 individuals/100 km^2^). Wolves exhibited significant habitat selection (χ^2^ = 23.6, *p* < 0.001), with 78% of detections occurring in alpine meadows and bare rocks at elevations of 4200–4300 m. The total wolf population was estimated to range from 2137 to 9169 individuals. Kernel density estimation (KDE) revealed a diurnal activity rhythm in wolves with minimal seasonal variation (Δ_4_ = 0.88). Peak activity intensity occurred between 8:00 and 15:00, and 17:00 and 21:00, during the cold season, and between 10:00 and 15:00, and 18:00–22:00, in the warm season. The study provides a scientific basis for more effective management and conservation, enhancing understanding of wolf populations in Qinghai and peripheral regions.

## Simple Summary

Understanding the population dynamics and behavior of large carnivores is important for protecting wildlife and maintaining healthy ecosystems. In this study, we focused on the Tibetan wolf in Dulan County, located on the Qinghai–Tibet Plateau. We used a large network of motion-sensing cameras set across the mountains to estimate how many wolves live there, what types of environments they prefer, and when they are most active during the day. We found that wolves mostly live in high mountain areas between 4100 and 4300 m, where the land is mostly rocky and covered with alpine grasslands. We estimate that about 2137 to 9169 wolves live in this region. These wolves are most active around sunrise and sunset, and this pattern stays the same throughout the year. Our findings help explain how wolves adapt to life in harsh mountain conditions and provide useful information to support efforts to protect their habitat and population in the future.

## 1. Introduction

Wolves (*Canis lupus*) are a wildlife species of significant scientific concern. Historically widespread across the Northern Hemisphere, it functions as an apex predator in ecosystems and exerts critical top–down regulatory effects [1,2,3,4]. In recent decades, the wolf’s global distribution has contracted dramatically, with populations declining continuously [5,6,7]. By the late 19th century, most wolf populations in Western Europe had been extirpated amid severe habitat fragmentation [8]; by the mid-20th century, wolves were nearly eradicated from the contiguous 48 U.S. states, surviving only in small numbers in northern Minnesota [9]. According to the research of Mooney [10], Ethiopian wolf populations have declined persistently over the past decade; surveys by South Korea’s National Institute of Biological Resources indicate the Korean wolf (*Canis lupus coreanus*) is considered extinct throughout the Korean Peninsula [11].

Wolves in China are mainly distributed in Xinjiang, Qinghai, and Inner Mongolia [12]. Currently, several studies on genetic evolution analysis, population distribution, habitat selection, and breeding behaviors of wolves in Xinjiang and Inner Mongolia have been conducted [13,14]. The Tibetan wolf (*Canis lupus chanco*) is a genetically distinct subspecies adapted to the Qinghai–Tibet Plateau and the Himalayas. It exhibits morphological and physiological adaptations to hypoxia, including gene selection such as RYR2 [12,14,15,16]. Diet studies from the northeastern Tibetan Plateau show a preference for wild ungulates like Tibetan gazelle, blue sheep, and woolly hare or pika, with limited livestock consumption where wild prey is available. Supplementary prey includes small mammals like marmots and hares [17]. Tibetan wolves have large home ranges, often exceeding hundreds of square kilometers, due to low prey density and the need to cover vast areas (territories up to ~2100 km^2^) [18]. Habitat selection favors open alpine steppe and meadow ecosystems above ~4000 m, avoiding areas with high human disturbance, roads, and settlements [15,19,20,21,22,23,24]. Additionally, the distribution and population size of wolves in these two provinces and Sichuan Province have been scientifically estimated [25,26,27]. However, the population status of wolves in Qinghai–Tibet Plateau remains largely unknown, where wolves’population density and spatial distribution, as well as activity rhythm, are all unclear.

Population size and density are foundational parameters in animal ecology, underpinning evidence-based conservation and guiding effective population management strategies [28,29,30]. Population estimation of species is conducive to the long-term conservation of populations. Spatial distribution and temporal rhythms are also critical population characteristics, representing the outcomes of wildlife’s long-term adaptation to natural survival [31,32]. These characteristics are influenced by numerous environmental factors. For example, vegetation and altitude affect the reciprocal migration of giant pandas (*Ailuropoda melanoleuca*) between different bamboo forests in the Meigu Dafengding Nature Reserve to obtain sufficient food [33]. Human disturbances cause vertical migration of Asiatic black bears (*Ursus thibetanus*) to higher elevations in Shennongjia to avoid roads and settlements [34]. These studies collectively demonstrate that the spatial distribution and activity patterns of wildlife are fundamentally shaped by survival strategies and evolutionary adaptations to environmental changes. Top predators often adjust their behaviors, such as diel activity patterns and habitat selection, to cope with resource fluctuations and human disturbances, thereby sustaining their ecological roles [35,36,37,38]. These studies collectively demonstrate that the spatial distribution and activity rhythms of wildlife reflect their survival strategies and evolutionary adaptations in response to environmental changes [39]. Considering the ecological importance of apex predators and the uniqueness and fragility of the Qinghai–Tibet Plateau [37,40,41,42,43,44,45,46,47], information on these population characteristics is urgently required for wolf conservation efforts in Qinghai.

In recent years, camera trap technology has rapidly developed and been widely applied in wildlife surveys [48,49,50,51,52,53,54]. Compared with traditional survey methods, this technology exhibits high concealment and causes minimal disturbance to wildlife [55]. This study utilized camera trap technology to monitor wolves in Dulan County, Qinghai Province, aiming to accurately assess the population size, spatial distribution, and activity rhythm of wolves in this region, thereby providing a scientific basis for conservation efforts.

## 2. Study Method

### 2.1. Study Area

**Figure 1:**
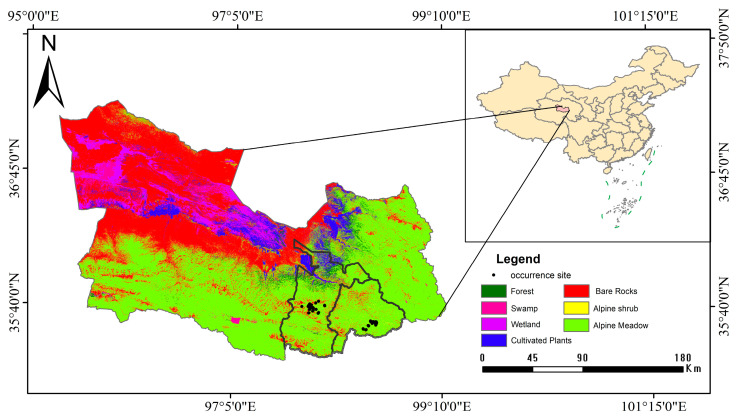
Map of study area and wolf distribution site in Dulan, Qinghai, China.

Dulan County, located in the southeastern Qaidam Basin, is recognized as one of Qinghai Province’s top ten resource-rich counties. The study area encompasses Gouli and Xiangjia Townships in Dulan County (Figure 1), spanning latitudes 35°20′–37°22′ N and longitudes 95°34′–99°40′ E (Figure 1), with a mean altitude of 3100 m. This region is characterized by a plateau arid continental climate, featuring an annual average temperature of 3.7 °C [56] and mean annual precipitation of 201.9 mm [57]. In the study area, the annual cycle was classified into cold (October–April) and warm (May–September) seasons [39,40,41]. Dominated by natural grasslands, the landscape exhibits a forest cover rate of 3.58% [58]. Primary habitats comprise alpine meadows (76.3% coverage), with fragmented distributions of alpine shrublands (18.1%) and scree slopes (5.6%) above 4000 m [59]. This region harbors high wildlife diversity, including emblematic species such as the Tibetan brown grizzly bears (*Ursus arctos pruinosus*), snow leopard (*Panthera uncia*), wolf, plateau pika (*Ochotona curzoniae*), blue sheep (*Pseudois nayaur*), and white-lipped deer (*Gervus albirostris*) [60].

### 2.2. Set up Camera Traps

From July 2021 to July 2022, camera traps were randomly deployed along valley axes, with perpendicular distances of 200–500 m from the valley centerline. A minimum inter-camera distance of 300 m was maintained to ensure spatial independence of sampling units. A total of 150 camera traps (CL-A1 manufactured by WildNature Tech Co., Ltd., based in Qingdao, China. More information on this model CL-A1 can be found on the company’s official website: www.yequziran.com, accessed on 7 August 2025) were deployed across the study area for 1 consecutive year (Table S1). Each unit was secured to rocks at a height of 40–80 cm above ground level and configured with medium sensitivity to capture two consecutive photographs per trigger event at five-second intervals. Spatial autocorrelation analysis was conducted using Moran’s I index in ArcGIS. The global Moran’s I index was 0.0652, with a *p*-value of 0.8802. All camera traps were equipped with infrared flash units to minimize disturbance to wildlife, and were passively triggered by passive infrared (PIR) motion sensors. No bait or olfactory attractants were used to alter animal movement or behavior. Geospatial parameters, including elevation, GPS coordinates (decimal degrees), and habitat classification, were systematically recorded for each camera station. Habitat types were classified using high-resolution remote sensing imagery combined with field verification. Multispectral satellite data (10–20 m resolution, acquired in summer 2021) from the National Tibetan Plateau Data Center were used to derive initial land cover types [61]. These were grouped into four habitat categories: alpine meadow, alpine steppe, shrubland, and barren land, using supervised classification. The results were subsequently validated and refined based on field observations at the deployment sites of 150 camera traps. This integration of remote sensing and ground-based data ensured accurate delineation of habitat boundaries and classification relevant to the ecology of the focal species.

### 2.3. Data Analysis

#### 2.3.1. Independent Captures

Photographs of the same species captured within one day at the same camera location were treated as a single detection event to prevent overcounting of individuals [62]. Photographs from the camera traps are renamed using ACDsee (e.g., “Dulan01-0001” to “Dulan01-0999”) and metadata such as timestamps is exported. Species are manually identified, and data, including species name, quantity, and date, are recorded in Excel. Finally, independent photo counts are calculated based on time intervals between photos, with wolves counted using a one-day interval to determine the final count.

#### 2.3.2. Estimate Population Size

A random encounter model [63] was used to estimate the population size and density of wolves. Formula for calculating density:

(1) $$D=\frac{y}{t}\frac{\pi }{vr\left(2+\theta \right)},$$

in which *D* is the population density of wolves, *y* is the number of independent photos, *t* is the investigation time (different values of *t* represent the total time during which camera traps are deployed at wolf encounter locations, with the duration varying across different habitats depending on the number of cameras.), π is a constant, *v* is the moving speed of wolves (in days), *r* is the radius of camera sector detection area, and *θ* is the included angle sector detection area (expressed by radian value rad).

The angle of view of the camera traps is 58° (*θ* = 1.01 rad), with a maximum detection distance of 20 m (*r* = 0.020 km). Limited data exist on the daily movement speed of wolves in Qinghai; however, some studies on the wolf activity in Alberta, Canada, report a maximum daily travel distance of 50.0 km for wolves [64,65], documenting an average daily movement speed of 33.55 ± 31.46 km/d, with pre-and post-snowfall speeds ranging from 11.30 km/d to 13.14 km/d. Based on these findings, we selected three speed parameters: 11.3 km/d, 33.6 km/d, and 50.0 km/d [66], Based on the three vegetation types in the study area, We selected three speed parameters: 11.3 km/day [67], 33.6 km/day [68], and 50.0 km/day [67] for estimating the wolf population density in Dulan County.

#### 2.3.3. Capture Rate

The relative abundance of wolves across different elevations and habitat types was assessed using capture rate (CR) as the key metric [69], calculated as follows:

(2) $$CR=\frac{Pi\times 100}{T},$$

where *Pi* is the number of independent photographs of wolves captured by camera traps in that habitat type, *T* is the total number of effective camera-trap days.

#### 2.3.4. Activity Rhythm

Nighttime was defined as the period from 20:00 to 6:00 the following morning, based on the actual sunrise and sunset times of the local area. The diel activity patterns of wolves in the cold and warm seasons were analyzed using the kernel density estimation (KDE) method [70,71]. We employed the overlap [71,72] and activity packages [70] in R Studio (R version 4.4.3) to generate KDE curves and calculate the overlap coefficient (Δ) to assess temporal niche overlap of gray wolves across different study areas within the same season. In the assessment of pairwise comparisons, the Δ_4_ coefficient is implemented when both sample sizes are ≥75; conversely, the Δ_1_ coefficient is employed should at least one sample size drops below 75 [73]. A Δ_1_ or Δ_4_ value of one denotes complete temporal overlap, while a value of zero indicates complete temporal segregation [74].

## 3. Results

Between July 2021 and July 2022, 150 camera traps were initially deployed throughout the designated research zone in Dulan County, Qinghai Province. Certain units malfunctioned or were lost, resulting in 114 functional cameras; these accumulated a total monitoring effort of 41,610 trap-days. Wolves were recorded at 68 distinct camera locations, representing 59.65% of all operational sites, yielding 392 temporally independent photographs of the species (Table S2).

### 3.1. Population Size

Based on field surveys in Dulan County, Qinghai Province, wolf activity was confirmed primarily across three habitat types: alpine scrub, alpine meadow, and bare rock. Population density estimates derived from the REM revealed significant spatial variation: 7.81 ± 1.31 individuals/100 km^2^ in alpine scrub, 4.87 ± 0.87 individuals/100 km^2^ in alpine meadow, and 21.39 ± 3.79 individuals/100 km^2^ in bare rock habitats. Given the total area of these habitats (45,272.5 km^2^), habitat-weighted density integration yielded an estimated total wolf population of 2137–9169 individuals. The estimated wolf population for the cold season was 2135 ± 5394, while the population density for the warm season was 1679 ± 4246. (The population density calculated for each habitat, based on the corresponding movement speed, was multiplied by the area of each respective habitat to estimate the total wolf population in the study area.).

### 3.2. Habitat Types and Altitude Preferences

Research indicates that wolves exhibit the highest capture rate in bare rock habitats (2.02), followed by alpine meadows (1.79), with the lowest rate observed in alpine scrub (1.29) (Table 1). The capture rate peaks at elevations between 4200 m and 4300 m (2.44), while the rate is minimal above 4300 m (Table 2).

### 3.3. Activity Rhythm

#### 3.3.1. Daily Activity Rhythm

**Figure 2:**
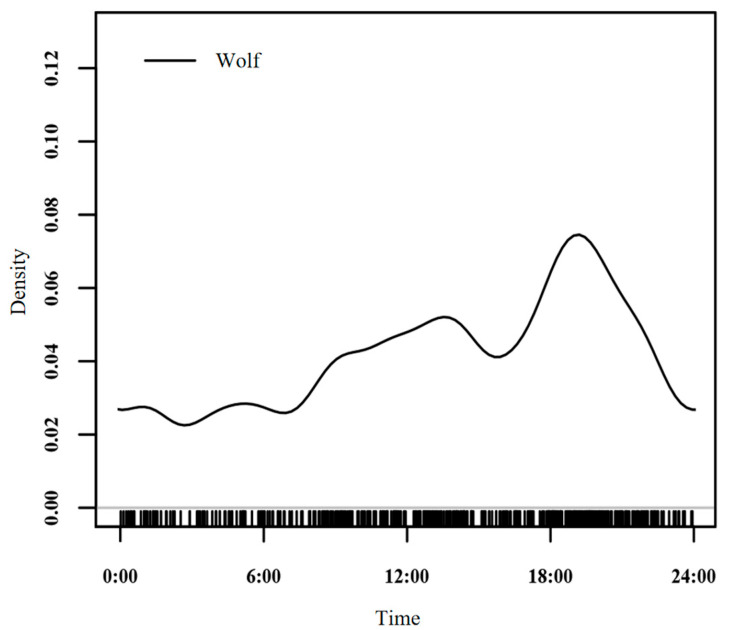
Daily activity rhythm curve of wolves in Dulan, Qinghai.

Wolves are diurnal animals (the kernel density area proportion during 6:00–20:00 accounts for 65%). Within a day, wolves exhibit two activity peaks: a midday peak at 13:00–15:00 and a dusk peak at 18:00–20:00. Activity intensity is relatively low during other time periods (Figure 2).

#### 3.3.2. Seasonal Variation in Activities

**Figure 3:**
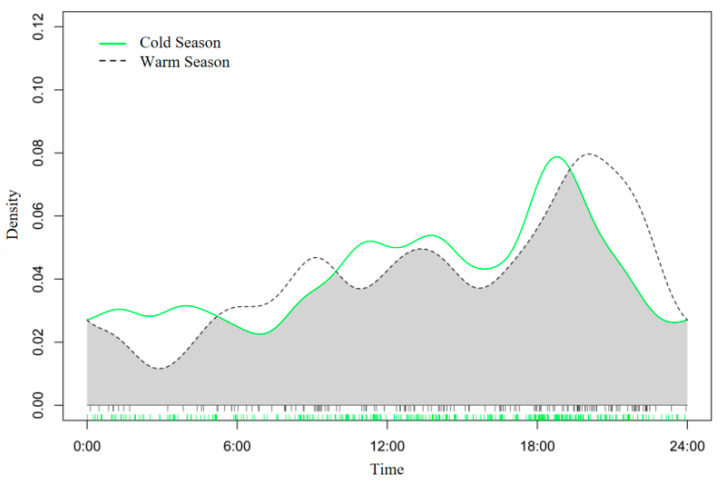
Diel activity rhythm of wolves across different seasons in Dulan, Qinghai. The shaded area represents the overlap of the activity rhythms of wolves during the cold and warm seasons.

During the cold and warm seasons, the numbers of independent valid captures of wolves were 281 and 150, respectively. The overlap coefficient of daily activity rhythms between seasons was Δ_4_ = 0.88 (Figure 3), indicating minimal seasonal variation in wolf activity patterns. The dusk activity peak occurred at 19:00 in the cold season and 20:00 in the warm season, suggesting a slightly earlier peak in the cold season.

## 4. Discussion

### 4.1. Population Denstiy

A total of 392 independent photos were captured by the camera traps, documenting 758 individual wolves. Due to the absence of video data, precise individual identification was not possible. Among these, only two independent photos featured two adults and two pups, and three independent photos depicted groups of more than ten individuals. The highest number of individuals in a single independent photo outside of group occurrences was eight. The majority of the photos captured solitary individuals, with 248 photos showing only one wolf. Overall, there was no clear pattern in the appearance of wolf groups.

Research methods for estimating the population density of large wildlife include distance sampling, mark-recapture, encounter-based methods, and remote sensing direct counting, all based on different estimation principles [75]. For camera trap surveys, distance sampling, mark-recapture, and encounter-based methods are commonly used [76]. In distance sampling, distance can be measured using field surveys (for studies with few camera sites) [77]; mark-recapture methods based on camera traps typically rely on unique body features for individual identification [78]. Camera trap-based mark-recapture methods typically rely on identifying individual animals based on unique body features [79], but wolves’ low distinctiveness in body features makes this method unsuitable for wolf density estimation in our study area [80]. Encounter-based models (e.g., random encounter, random encounter and staying time, time-to-event, space-to-event, and occupancy models) do not require assumptions about home ranges or individual identification [76]; since the camera traps used here lack video capabilities, estimating species’ dwelling time is not possible, making time-dependent models inapplicable. Occupancy models, including the Royle–Nichols model, assume clearly defined home ranges [81]. But wolves in Qinghai exhibit dispersed and non-fixed activity patterns during non-breeding seasons [82,83]; therefore, the random effects model (REM), which assumes random wildlife movement within the study area, best matches the observed movement patterns of wolves. REM provides more accurate density estimates in the absence of individual identification and video data, making it the most suitable method for this study.

As an apex predator in alpine ecosystems, wolves positively contribute to maintaining ecosystem health by exerting top–down regulation on food chains, thereby influencing species composition and community structure. This study represents the first systematic investigation of wolf populations in Dulan County. Compared to wolf population densities in Galicia, Spain (2.88 ± 0.37 individuals/100 km^2^) [84], Arezzo Province, Italy (1.21 ± 0.27 individuals/100 km^2^) [85], and Inner Mongolia’s Saihanwula National Nature Reserve (4.18 ± 2.88 individuals/100 km^2^) [25], the wolf population status in Dulan County (4.87 ± 0.87 to 21.39 ± 3.79 individuals/100 km^2^) is relatively healthy. Wolf population density is influenced by multiple factors, such as food resources, habitat conditions, predation pressure, and anthropogenic interventions [86,87,88]. In Dulan County, alpine meadows and rocky outcrops provide abundant food sources for wolves, such as livestock and small mammals, which constitute their preferred prey [89]. From the camera trap images, it can be seen that the distribution of wolves in Dulan County overlaps with human agricultural activity areas to some extent [90,91]. Wolves in Dulan County primarily inhabit high-altitude areas characterized by sparse human populations and relatively low anthropogenic disturbance, which likely contributes to their relatively healthy population status.

The estimated wolf population for the cold season was 2135 ± 5394, while the population density for the warm season was 1679 ± 4246. The considerable difference in population density between the two seasons remains unexplained, with several possible contributing factors. One hypothesis is that wolves may aggregate in specific areas during the cold season, potentially due to resource availability or social dynamics [92,93,94]. Additionally, Dulan County may function as a key hotspot for wolf activity during the cold season, which could contribute to the observed increase in population density. To further investigate this discrepancy, we recommend expanding the camera trap coverage and monitoring duration in future studies, allowing for a more comprehensive understanding of seasonal variations in wolf population dynamics [92,95].

Further research could also explore how environmental factors, such as habitat preferences and prey availability, might influence wolf aggregation patterns across seasons. The interplay between ecological conditions and anthropogenic disturbance may also provide insight into the observed trends.

Although this study provides an initial estimate of wolf population density on the QinghaTibet Plateau, there are several limitations. Firstly, while camera trap technology offers an efficient monitoring tool, the relatively close placement of the cameras may lead to non-independence of samples. Although we attempted to address this issue by using the random encounter model (REM), this limitation still requires further optimization in future studies. To mitigate this effect, future research could consider increasing the distance between cameras or incorporating other remote sensing technologies for more comprehensive monitoring.

### 4.2. Habitat Type and Elevational Preferences

Wolves are widely distributed throughout Xiangjia township, Gouli township, as evidenced by detection in 59.65% of deployed camera traps; however, significant distributional variations occur across habitat types and elevational gradients. This heterogeneity is likely attributable to differential habitat provisions of concealment conditions and food resources [96], as well as varying detection probabilities across different habitat types. For example, dense shrublands may pose greater challenges for camera detection, potentially leading to underrepresentation of wolves in such areas [97,98]. Bare rock habitats exhibit the highest detection rates, potentially due to reduced anthropogenic disturbance and superior camouflage opportunities [25]; Alpine meadows show the second-highest detection frequency, primarily because they sustain abundant herbivore prey populations that indirectly govern predator distributions [99]. Wolf distribution peaks within the 4100–4300 m elevational band, with notably lower densities in lower (<4100 m) and higher (>4300 m) zones. Lower elevations experience heightened anthropogenic interference [100,101], while higher elevations suffer from vegetation scarcity and persistent snow cover that diminish prey availability [102]. The optimal 4100–4300 m range coincides with extensive alpine meadows providing consistently rich food resources, reinforcing the elevation-dependent distribution pattern observed.

### 4.3. Activity Rhythm

For most previous reports, wolves have been predominantly classified as nocturnal in most studies [103,104,105,106,107,108], In regions with apex predators, including wolves, temporal partitioning rather than spatial overlap allows coexistence, with wolves adjusting their activity patterns to minimize competition with other large carnivores like cougars and brown bears, often driven by habitat preferences and resource availability [109,110,111,112,113]. Populations in Dulan County exhibit marked diurnal activity patterns. This behavioral shift may serve to mitigate competition with sympatric nocturnal large carnivores, including brown bears, Eurasian lynx, and snow leopards [114,115]. Additionally, nocturnal constraints arise from the extreme thermal conditions of the plateau, particularly during cold seasons when temperatures can plummet to −30 °C [116], further limiting activity during nighttime hours.

The activity rhythm exhibits a bimodal pattern, consistent with patterns observed in other diurnal mammals [117]. While most species rapidly reach their first activity peak shortly after sunrise, wolves in Dulan County display a gradual increase in activity intensity post-sunrise, peaking in the early afternoon. This delayed initial peak may stem from slower thermal inertia under low temperatures [118,119]. The second overlapping activity peak occurs between 19:00 and 20:00; the late emergence of the first peak results in a shorter inter-peak resting interval. Seasonal variation in wolf diel activity rhythm is minimal, with high overlap coefficients across seasons. The sole distinction manifests as activity peaks occurring approximately one hour earlier during cold seasons—a likely adaptation to earlier nightfall in winter.

The wolf population in Dulan County, Qinghai Province, maintains a relatively healthy status, though its abundance may be influenced by prey availability. Strengthening protection of alpine meadows is critical to securing suitable habitats. Human activities, particularly livestock grazing, may induce spatial displacement during breeding seasons, highlighting anthropogenic disturbance as a key factor affecting population density. Implementing regulated grazing practices is recommended to mitigate human–wolf conflicts. Diurnal activity rhythms exhibit a bimodal pattern primarily driven by interspecific competition and thermal constraints, with minimal seasonal variation. Establishing long-term population monitoring is essential to track spatiotemporal dynamics and demographic trends. These findings aim to enhance ecological understanding of Qinghai wolves and provide theoretical frameworks and empirical baselines for wildlife authorities to develop science-based conservation management.

## 5. Conclusions

This study provides the first systematic assessment of the population density, spatial distribution, and activity rhythm of Tibetan wolves (*Canis lupus*) in Dulan County, Qinghai Province, using a camera trap trapping framework combined with the random encounter model. Our results indicate that wolves are widely distributed across alpine habitats, particularly in bare rock and meadow environments between 4100 and 4300 m, with an estimated population ranging from 2137 to 9169 individuals. The diel activity rhythm of wolves exhibited a diurnal bimodal pattern with minimal seasonal variation, likely driven by thermal constraints and interspecific interactions.

These findings fill a critical data gap in understanding the ecology of wolves on the Qinghai–Tibet Plateau and provide an empirical foundation for science-based conservation strategies. Future efforts should focus on maintaining habitat connectivity, regulating anthropogenic disturbances in core habitats, and implementing long-term monitoring programs to ensure the continued stability of wolf populations in this ecologically sensitive region.

## Figures and Tables

**Table 1:** Capture rate of wolves in different habitat types in Dulan, Qinhai.

Habitat	Number of Independent Photos	Number of Cameras	Number of Camera Days	Capture Rate
Alpine shrub	236	14	5110	1.29 ± 0.3
bare rocks	92	14	5110	2.02 ± 0.5
alpine meadow	64	40	14,600	1.79 ± 0.5

**Table 2:** Capture rate of wolves in different altitudes in Dulan, Qinghai.

Altitudes	Number of Independent Photos	Number of Cameras	Number of Camera Days	Capture Rate
<4000 m	51	11	4015	1.39 ± 0.5
4000–4100 m	155	26	9490	1.77 ± 0.4
4100–4200 m	68	8	2920	2.40 ± 0.8
4200–4300 m	91	12	4380	2.44 ± 1.6
4300–4400 m	25	10	3650	0.74 ± 0.3
>4400 m	2	1	365	0.82 ± 0

## Data Availability

The data presented in this study are not publicly available due to confidentiality restrictions. Requests for data access should be directed to the corresponding author.

## Supplementary Materials

The following supporting information can be downloaded at: https://www.mdpi.com/article/10.3390/biology14091273/s1, Table S1. Infrared Camera Deployment Sites in Dulan, Qinghai. Table S2. Independent Photographs of Wolves in Dulan, Qinghai.
